# Total organic carbon (TOC): a simple tool for assessing micro(nano)plastics and nanocellulose recovery during size-based fractionation

**DOI:** 10.1007/s00216-025-05812-4

**Published:** 2025-05-19

**Authors:** Guillaume Bucher, Gabriella F. Schirinzi, Chiara Verra, Hind El Hadri, Otmar Geiss, Douglas Gilliland

**Affiliations:** European Commission, Joint Research Centre (JRC), Ispra, Italy

**Keywords:** Microplastics and nanoplastics, Nanocellulose, Recovery, AF4, TOC, SPES

## Abstract

The assessment of analyte recovery during sample preparation is a critical quality control parameter in method development. While elemental mass spectrometry techniques, such as ICP-MS, are very effective for assessing the recovery of particulate materials containing metallic elements, there is no equivalent applicable to metal-free carbon or CHNO-based particulate polymer materials. Vibrational spectro-microscopy or thermo-analytical techniques can be used to quantify polymer-based micro- and nanoparticles, but are typically expensive and time-consuming techniques that require higher levels of expertise. This study investigated the potential of a liquid-based total organic carbon (TOC) analyzer as a simple, cost-effective, and universal method for determining the recovery of polymer-based particulate micro- and nanomaterials following filtration, centrifugation, and asymmetric flow field flow fractionation (AF4) processes. A good correlation between solid contents and TOC analysis was demonstrated for standard polystyrene (PS) particle suspensions of various sizes, ranging from 50 nm to 90 μm (79.2 to 113.6% recovery), and other types of synthetic and natural polymeric particle suspensions, including polyethylene (PE), polypropylene (PP), polyvinyl chloride (PVC), polyethylene terephthalate (PET), and cellulose (86.2 to 126.2% recovery). Liquid-based TOC was then successfully applied to estimate particle recovery after various preparatory and fractionation steps, including the determination of filtration recoveries for nanocellulose suspensions (99.0 to 101.4% recovery) and PS micro- and nanoparticles spiked into environmental lake and river freshwater samples (70 to 96% recovery). The combination of TOC and single particle extinction and scattering (SPES) measurements allowed the tracking and quantification of three different populations of PS particles in a mixture (200, 500, and 1000 nm) during successive centrifugation steps (113.8 ± 13.9% cumulative recovery). Finally, this study demonstrated the suitability of TOC for determining both the absolute and relative recoveries of polymer-based particulate materials after AF4 fractionation in line with ISO standards. Liquid-based TOC proved to be a valuable tool for directly tracking, quantifying, and evaluating the recovery of polymer-based micro- and nanoparticles in model and environmental water samples before and after routine size-based fractionation steps.

**Graphical abstract:**

Graphical abstract created in BioRender (https://BioRender.com/u57r657)

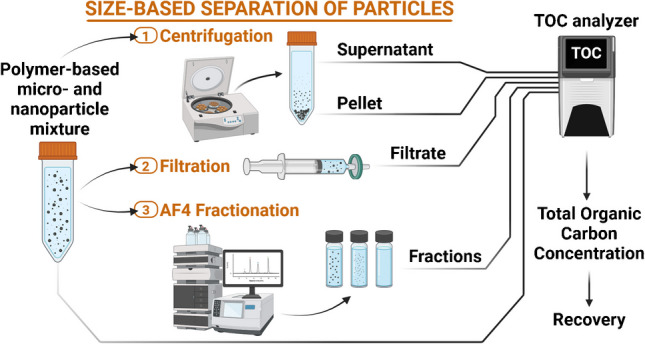

**Supplementary Information:**

The online version contains supplementary material available at 10.1007/s00216-025-05812-4.

## Introduction

Lately, there has been a growing interest in the literature for the measurement of particulate organic matter, including microplastics, sewage particles, and algae, in various environmentally relevant matrices, including sediments, freshwater, seawater, and wastewater by using a total organic carbon (TOC) analyzer [1–8]. TOC analyzers are capable of directly measuring the total carbon (TC) content and the total inorganic carbon (TIC) content of a sample (liquid or solid). The TOC content is therefore indirectly measured as the difference between TC and TIC as TOC = TC − TIC. Alternatively, TOC can be measured as non-purgeable organic carbon (NPOC) following in situ sample pretreatment to remove carbonates prior to TC measurement [6, 8, 9]. TOC is commonly employed for determining the extent of organic content in water, which in turn serves as an important indicator of the general quality of water [10–12]. It is important to note that the TOC in liquid samples includes both the dissolved organic carbon (DOC) and the particulate organic carbon (POC). The limit between DOC and POC is commonly set by the well-established 0.45 μm filtration cut-off [10, 13, 14].

When focusing on microplastics and polymeric materials (e.g., cellulose), some studies demonstrated correlations between TOC and microplastic content in samples such as tap water, river water, seawater, and wastewater [2–5]. However, most of the published protocols relied on a specific set of sample treatments (e.g., filtration, Fenton digestion, density separation, drying) that ultimately resulted in a solid residue, which was then analyzed using a TOC analyzer setup for solid samples. With a detection limit as low as 0.01 to 0.1 µg organic carbon, the use of a TOC analyzer setup for liquid samples may become advantageous, especially for direct analysis of relatively clean water samples containing micro- and nanoplastics. Unfortunately, liquid-based TOC can become problematic when the Brownian motion of the particles in solution becomes negligible compared to gravitational sedimentation. Indeed, the absence of a re-dispersion functionality in the sample injection system of a liquid TOC analyzer would inevitably lead to particle losses and low recovery rates due to settling and/or floatation [7]. That being said, many instruments are in fact equipped with re-dispersion functionality and tubing, which theoretically allows for the introduction of particulates up to approximately 700 μm.

The objective of this study was to investigate the potential of liquid-based TOC as an analytical tool to track and/or quantify carbon-based particulate materials (e.g., microplastics, nanoplastics, and nanocellulose samples) during the development of sample preparation methodologies. In this context, TOC analysis was optimized and applied to determine and check the solid content of various types of aqueous nano- and microplastics suspensions with particle sizes ranging from 50 nm to 90 μm. The performance of the TOC instrument was primarily tested using polystyrene (PS) standards, a key reference material in the field of micro- and nanoplastics. PS standards are widely used in method development due to their monodispersity, stability, and availability in high concentrations. Additionally, TOC analysis was tested for its applicability to evaluate the efficiency after size separation by centrifuge, filtration and fractionation. The results are discussed in terms of the applicability of the liquid-based TOC approach, taking into account size limitations and potential interferences.

## Material and methods

### Instrumentation

TOC determinations were performed with a TOC-L CPH instrument from Shimadzu (Kyoto, Japan) setup for liquid samples and equipped with high sensitivity catalyst and pure oxygen (O_2_) as carrier gas. Fifty-milliliter polypropylene (PP) centrifuge tubes with PP cap from Corning (Corning, NY, USA) were rinsed three times with ultrapure water and allowed to dry prior to use for TOC analysis. This rinsing step was introduced as a precautionary measure to avoid unstable background caused by the possible presence of residual volatile organic compounds (e.g., plasticizers, unreacted monomers) in the capped PP centrifuge tubes.

Cellulose nanocrystal and nanofibers samples were dispersed with a VCX130 probe sonicator (130 W, 20 kHz) from Sonics (Newtown, CT, USA) equipped with a Ti-6Al-4 V 3 mm alloy probe, then filtered with Millex-HV 0.45 μm PVDF syringe filters from Millipore (Burlington, MA, USA). Filtrates were analyzed with an Asymmetric Flow Field Flow Fractionation (AF4) Eclipse Dualtec separation system (10 kDa regenerated cellulose membrane and 350 μm spacer) coupled to a DAWN 8 + HELEOS II multi-angle light scattering (MALS) detector and an Optilab refractive index (RI) detector all from Wyatt Technology (Santa Barbara, CA, USA).

Solid content measurements and gravimetric dilutions were performed using an AX204 analytical balance and a XPR2U micro-balance from Mettler Toledo (Columbus, OH, USA).

Other sample preparation and analysis equipment included a 5804R centrifuge from Eppendorf (Hamburg, Germany) equipped with a swing bucket rotor A-4–44, a DU-32 digital ultrasonic cleaner (120 W, 40 kHz) from Argo Lab (Carpi, Italy), a RET basic magnetic stirrer from IKA (Staufen, Germany), and a Single Particle Extinction and Scattering (SPES) Classizer™ ONE system from EOS S.r.l. (Milan, Italy).

### Chemicals, reagents, and environmental samples

Sodium carbonate (Na_2_CO_3_, 11.33%C), potassium hydrogen phthalate (KHP, C_8_H_5_KO_4_, 47.05%C), and phosphoric acid (H_3_PO_4_, 85% wt) were from Sigma-Aldrich/Merck (Darmstadt, Germany), and ultrapure water (UPW) 18.2 MΩ cm was from Millipore (Burlington, MA, USA) production system.

Commercially available micro- and nanoplastic particles in aqueous suspension were from Polysciences (Hirschbergan an der Bergstrasse, Germany) and Sigma-Aldrich/Merck (Darmstadt, Germany) (detailed references in Table S1). Au-doped PE, PP, and PVC particles were synthesized in-house at the JRC (Cassano et al*.* 2021 and 2023, detailed references in Table S1) [15, 16].

Nanocellulose samples CNC 1 and NFC 1 were from the University of Maine (UMaine, Orono, ME, USA), CNC 2 from CelluForce (Québec, Canada), and CNC 3 from Nanografi (Jena, Germany) (detailed references in Table S1).

Environmental surface water samples were collected from Lake Maggiore (Ispra, Italy, Coordinates 45°48′23.8″N–8°36′23.7″E) and the Variola River (Monte Ossolano, Italy, Coordinates 46°07′31.2″N–8°13′29.3″E). The concentrations of 39 elements along with the pH and total inorganic carbon (TIC) content of the environmental surface water samples are reported as supplementary information (Table S2).

### Methods

#### TOC measurements

Total organic carbon (TOC) content was calculated as the difference between total carbon (TC) content and total inorganic carbon (TIC) content (TOC = TC − TIC). In our case, TC and TIC determinations relied on the nondispersive infrared (NDIR) detection and quantification of CO_2_ gas released by (i) the chemical (acid) degradation of carbonated species for TIC and (ii) the catalytic (heat + Pt-based catalyst) degradation and oxidation of carbon-containing species (e.g., organic molecules, solvents, organic matter, micro- and nanoplastics, polymers, macromolecular organic compounds, and carbonated species) for TC. Overall calibration ranges were 0.01 to 10 mg organic carbon L^−1^ for TC (using KHP standard solutions and auto-dilution feature of the instrument) and 0.02 to 5 mg inorganic carbon L^−1^ for TIC (using Na_2_CO_3_ standard solutions and auto-dilution feature of the instrument). Injection volumes were automatically adjusted by the instrument depending on the carbon concentration ranges (Table 1). First injection was performed with the lowest injection volume (i.e., 50 μL for TC and 130 μL for TIC) to estimate the carbon content. Then, the instrument automatically selects the most appropriate volume of injection according to the carbon content to measure accurately TC and TIC. Samples were appropriately and gravimetrically diluted to fall within one of the TC ranges (usually aiming for 5 mg L^−1^ with TC Curve 3) and avoid further auto-dilution by the instrument. For each sample, TC and TIC were measured twice. A third measurement was automatically performed if the coefficient of variation (CV) of the two first measurements was > 2%. The final result for TC and TIC was the average of the two closest replicates in terms of TC or TIC content, the third replicate (if any) was rejected by the software. The multiple injection option was deactivated in order to avoid particle settling in the sampling syringe between replicate injections of a given sample. Vials containing samples with particle sizes above 1 μm were continuously agitated with a magnetic stirrer to avoid particle settling prior to aspiration into the TOC instrument.

**Table 1:** Injection volumes with respect to carbon concentration ranges

	Particle size	Volume of injection (μL)	Concentration range (mg L^−1^)	Calibration curve no
TC measurement	< 10 μm	2000 (max.)	TC from 0.01 to 0.1	TC Curve 1
		408	TC from 0.1 to 1.0	TC Curve 2
		50	TC from 1.0 to 10.0	TC Curve 3
TIC measurement	Not Applicable	2000 (max.)	TIC from 0.02 to 0.2	TIC Curve 1
		130	TIC from 0.2 to 5.0	TIC Curve 2
Modified TC measurement	> 10 μm	500	TC from 1.0 to 50.0	TC Curve 4

A modified TOC measurement procedure (affecting only the TC measurement while the TIC measurement remained unchanged) was applied to particles larger than 10 μm for which the colloidal behavior is negligible or completely lost [17, 18]. Therefore, for this modified TC method, the injection volume was increased and fixed at 500 μL and the calibration range was extended to 50 mg L^−1^. The sample vial was continuously agitated with a magnetic stirrer and any dead time in the syringe was eliminated either by setting fully independent injections (instead of the automatic replicate injection procedure) and/or by adding a mixing step of the syringe content prior to analysis to minimize any sedimentation/settling/floatation of particles.

Environmental surface water samples were analyzed by TOC: (1) as-is (unfiltered), (2) after filtration at 0.45 µm (typical dissolved/particulate threshold), (3) after spiking unfiltered water with 1 mg L^−1^ PS 1000 nm and 1 mg L^−1^ PS 50 nm, and (4) after spiking unfiltered water with 1 mg L^−1^ PS 1000 nm and 1 mg L^−1^ PS 50 nm and subsequent filtration at 0.45 µm to remove PS 1000 nm. In parallel, ultrapure water (UPW) was prepared and analyzed the same way to serve as a reference sample.

#### Centrifugal fractionation of polydisperse PS particle mixtures and SPES analysis

Centrifugation was used to separate a mixture of three commercially available PS beads according to their size, following Stokes’ law. TOC and SPES analysis were performed before and after different centrifugation steps. For this purpose, a mixture containing 5 mg L^−1^ each of PS 200, PS 500, and PS 1000 nm was prepared to assess the size separation efficiency of the centrifugation method on a simplified polydisperse sample. Eight milliliters of the equimassic PS mixture (total concentration of 15 mg L^−1^) was placed in a 10-mL glass vial, and 1 mL was collected for TOC and SPES analysis prior to centrifugation. The suspension was then centrifuged at 2500 RCF (3727 rpm) for 14 min (C1—1000 nm theoretical cutoff). The theoretical cutoff value is calculated according to Stokes’ law and means that only particles larger than this value have enough time to reach the bottom of the tube and form the pellet when starting from the top of the liquid column. The resulting pellet (F1 ≈ 0.5 mL) was collected, adequately diluted in ultrapure water, and measured by TOC and SPES. The supernatant (≈ 6.5 mL), which may contain a majority of nanoscale particles, was centrifuged again for 30 min at 4000 RCF (4714 rpm) (C2—500 nm theoretical cutoff). The subsequent pellet (F2 ≈ 0.5 mL) and supernatant (F3 ≈ 6 mL) were collected, diluted, and measured by TOC and SPES. All experiments were performed in triplicate.

SPES characterizes the way laser light is scattered by single particles in solution and provides a means to quantify and classify individual particles based on size and material specific optical properties. When particles pass through the focal region of a light beam, they transmit and scatter light, which is then collected by a sensor positioned in the far field of the laser beam [19, 20]. This configuration makes it possible to obtain information on the population of particles in a size range between 250 nm and 5 µm, including their refractive index, size distribution, and number concentration. This information can be obtained from the entire sample or a selected subset, depending on their characteristics. SPES data were acquired with an EOS Classizer™ ONE unit equipped with a red light diode (*λ* = 640 nm, power < 50 mW). The instrument operated at a constant flow rate of 4 mL min^−1^. The total analyzed volume ranged from 20 to 30 mL, with the option to recirculate the sample through the system to ensure the detection of sufficient particles. For the experiments in this section, the 500 and 1000 nm populations detected by SPES in each sample before and after centrifugal separation were isolated in order to measure the number-based concentration of these particles. Analyses were performed using Classizer™ SW1.4.42 software (EOS S.r.l., Milan, Italy).

For comparison purposes with TOC data, the number-based concentrations obtained from SPES were converted to mass-based concentrations taking into account particle sizes and a density of 1.05 g cm^−3^ for PS.

#### Nanocellulose fractionation by AF4-MALS-RI

Nanocellulose sample preparation and AF4 fractionation were adapted from Mukherjee and Hackley, 2018 [21] (see details in Table S3). In brief, nanocellulose standards were gravimetrically diluted in water to reach a concentration of 2% wt (for CNC 1 to 3) or 0.3% wt (for NFC 1). The diluted samples were then dispersed using a probe sonicator (12.5 W for 9 min) until reaching a total energy of 7 kJ and filtered using 0.45 μm PVDF syringe filters. When necessary, an additional dilution step was performed to reach a final concentration of 0.3% wt for all materials prior to injection in AF4. The injection volume was 50 μL, corresponding to 150 μg of cellulose. Mobile phase was 1 mM NaCl. Samples were injected both with and without crossflow in order to assess recovery with the RI detector. In both cases (i.e., with and without crossflow), fractions of interest (i.e., the void peak—F1 and the elution peak—F2, see Fig. S1) were collected in rinsed and tared 50 mL PP tubes for TOC analysis.

## Results and discussion

### Determination of micro- and nanoplastic content in suspensions

#### Determination of particle content in polystyrene standard suspensions

Commercial micro- and nanoplastic standards are usually provided with a certificate of analysis (CoA) specifying a “solid content.” This solid content serves as a proxy to the particle content and is usually expressed as a mass- or volume-based concentration or percentage. The idea was here to investigate the correlation between the solid content (provided on CoA or measured in-house, Table S1) and the TOC analysis taking into account the specific weight percentage of carbon present in the polymer (i.e., 92.26%C for PS).

In the literature, the determination of the TOC directly from liquid samples is commonly performed using the non-purgeable organic carbon (NPOC) method [6, 8, 9]. The NPOC method consists of a single injection of the liquid sample with in situ pretreatment by addition of HCl and sparging with carrier/combustion gas in the sampling syringe to eliminate the carbonates (i.e., TIC = 0 after pretreatment) and the purgeable organic carbon (e.g., volatile non-water miscible organic species) prior to injection on the catalyst for TC determination. In absence of purgeable organic carbon in the sample, the NPOC and TC-TIC methods should theoretically provide the same TOC value. These two methods (NPOC and TC-TIC) were preliminarily investigated with different PS micro- and nanoplastic suspensions. Overall, during the preliminary study, the recoveries for PS particles with sizes ranging from 200 nm to 30 µm ranged from 5 to 95% for the NPOC method and from 77 to 101% for the TC-TIC method. Possible cause for the observed discrepancies between NPOC and TC-TIC recoveries included the accelerated sedimentation and loss of larger particles due to their agglomeration/aggregation upon addition of HCl during the NPOC measurement process. From this point on, the investigation was continued using the TC-TIC method. Despite the necessity to perform two injections (i.e., one for TC determination and one for TIC determination), the TC-TIC method has the advantage of providing the TIC information, which can be useful in the characterization of environmental samples. Also the TC-TIC method does not require the optimization of HCl addition or sparging time, which are required for the NPOC method.

**Fig. 1:**
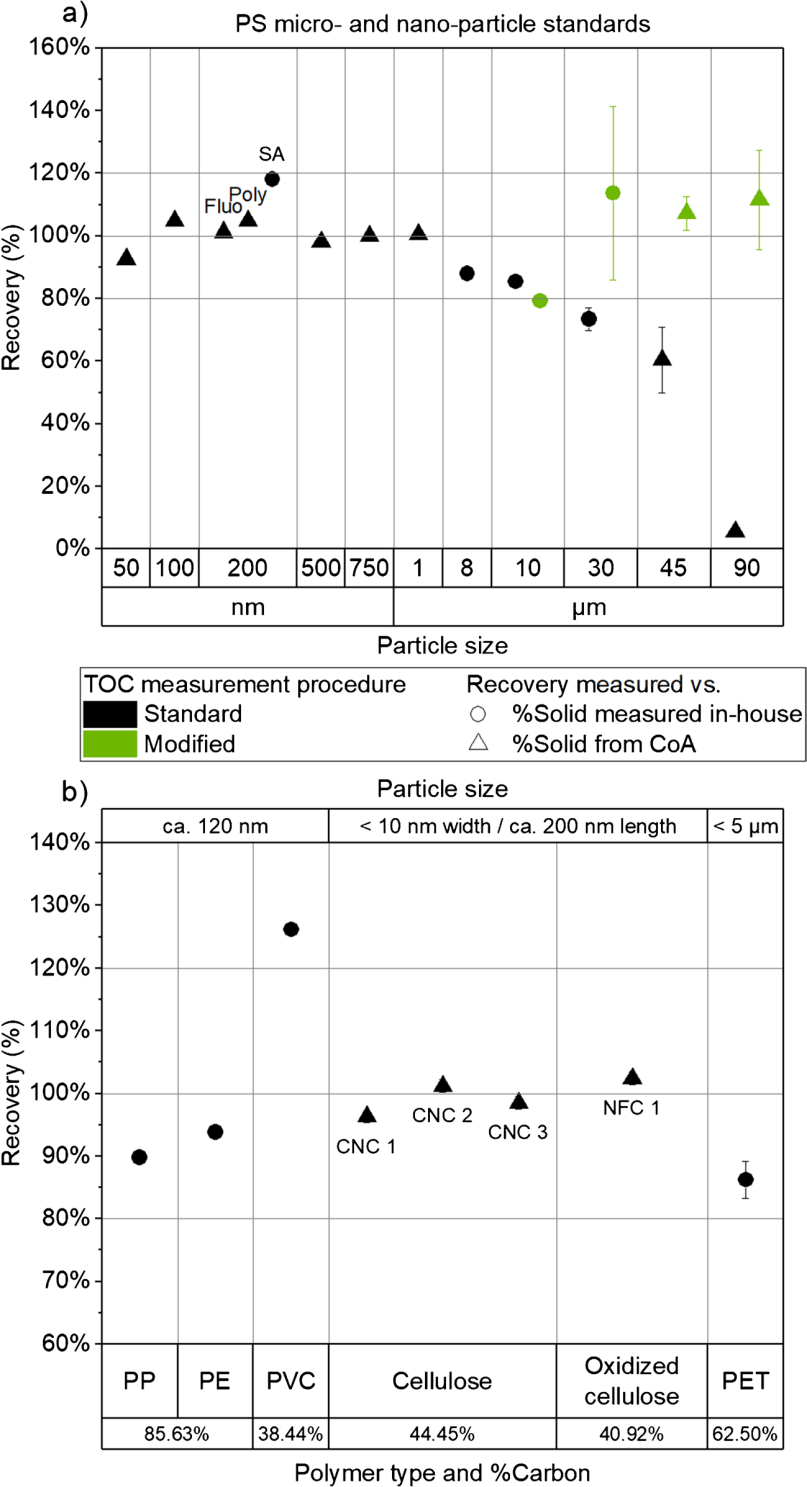
Recoveries expressed as the ratio between the solid content measured using the standard (black) or modified (green) TOC measurement procedure and the expected solid content provided on the manufacturer’s CoA (triangles) or measured in-house (circles) for **a** PS standards and **b** other types of polymers with their associated carbon weight percentages for information. Horizontal lines at 80%, 100%, and 120% added as visual guides. Fluo, Poly, and SA correspond to different manufacturers or types of 200 nm PS particles

Figure 1a and Table S4a present the recoveries expressed as the ratio between the particle content measured using the standard or modified TOC measurement procedure, derived from the TC-TIC measurement, and the expected solid content provided on the manufacturer’s CoA or measured in-house.

More specifically, Fig. 1a shows that the recoveries measured using the standard TOC measurement procedure and the expected solid content provided on the manufacturer’s CoA were very satisfactory, with low variability, and ranged from 92.6 to 104.9% for PS particles with different sizes ranging from 50 nm up to 1 μm. Good recoveries (i.e., 87.9% and 85.4%, respectively) and low variabilities, were also obtained for 8 μm and 10 μm PS particle suspensions for which the expected solid content was measured in-house.

For particles larger than 1 μm, a decrease in recovery was observed using the standard TOC measurement procedure, which became significant (less than 80% and down to 5.6% for PS 90 µm) for PS particles larger than 10 µm (with an increase in variability), requiring several modifications to the measurement procedure. This decrease in recovery was primarily attributed to the fact that colloidal behavior typically becomes negligible above 1 µm [17, 18] and particles are more susceptible to sedimentation and loss. However, this 1 µm threshold remains arbitrary and depends on the characteristics of each particle. In fact, recoveries obtained for 8 µm and 10 µm PS particles with the standard TOC measurement procedure were acceptable and well within the 80 to 120% range. In addition, the mass of spherical PS particles increases to the power of three in proportion to their size. Consequently, as shown in Table 2, PS particles larger than 10 µm become relatively heavy and only a few particles (e.g., theoretically 18 particles for 30 µm PS particles) are injected during TC measurement using the standard procedure. The modified TOC measurement procedure allows for increased injection volume, sample agitation, and calibration range.

**Table 2:** Theoretical sedimentation speed and number of PS particles injected in the TOC instrument during TC analysis using standard and modified procedures as a function of particle size

Particle size (μm)	Particle mass^a^ (μg)	Sedimentation speed^a,b^ (cm min^−1^)	Theoretical number of particles injected in the TOC	
			Standard procedure^c^	Modified procedure^d^
1	5.07 × 10^−7^	0.00016	492,663	19,706,540
10	5.07 × 10^−4^	0.016	493	19,707
30	1.37 × 10^−2^	0.15	18.2	912
45	4.62 × 10^−2^	0.33	5.4	270
90	3.70 × 10^−1^	1.32	0.7	33.8
150	1.86	3.68	0.1	6.7
300	14.8	14.7	0.02	0.8

^a^Considering particles as perfect hard spheres of polystyrene with a density of 1.05 g cm^−3^

^b^According to Stokes’ law in water at 20 °C and under standard acceleration of gravity (9.81 m s^−2^)

^c^Standard TC measurement procedure: aim 5 mg L^−1^ TC with 50 μL injected

^d^Modified TC measurement procedure: aim 25 mg L^−1^ TC with 500 μL injected

As supported by the theoretical data in Table 2, the modifications were motivated by (i) the lower number-based particle concentrations, (ii) the higher particle masses, and (iii) the faster sedimentation processes occurring with these micrometric PS particles. Therefore, in the modified TOC measurement procedure, the issue of lower number-based particle concentrations was addressed by increasing and setting the injection volume at 500 μL. Indeed, increasing the injected volume increased the probability of injecting a sufficient number of particles onto the catalyst (see Table 2). The poor recovery obtained for 90 µm PS particles (i.e., 5.6%) using the standard TOC measurement procedure indicates in fact that no particle was injected and detected by the system. As shown in Table 2, the standard TC procedure has a roughly 50/50 chance of injecting either zero or one 90 µm PS particle. Taking into account that the standard TC procedure does not include an agitation step prior to injection, the probability to inject one single particle becomes even smaller. The TOC measurement in this case most likely corresponds to traces of dissolved organic species (e.g., surfactant) initially present in the 90 µm PS standard as the dilution factor applied for the analysis of this standard was rather low compared to other PS standards. The introduction of the modified TOC measurement procedure allowed the injection of enough 90 µm PS particles (ca. 34 particles) to assess the particle recovery in the 80 to 120% range with a relative standard deviation of less than 15%.

The issue of higher particle masses was addressed by increasing the calibration range to 50 mg L^−1^ for TC while using the fixed 500 μL injection volume. Indeed, larger particles (combined with larger injection volume) are associated with larger amounts of carbon and therefore required a higher calibration range.

Finally, the issue of faster sedimentation processes was addressed by eliminating all dead time in the syringe (i.e., by setting three fully independent injections instead of the automatic duplicate/triplicate injection procedure) and/or by adding an automatic mixing step of the syringe content prior to analysis (i.e., suspension mode). Indeed, even after deactivating the “multiple injection” option, the automatic duplicate/triplicate injection procedure would pre-fill the syringe between injections 1–2 and 2–3, resulting in a dead time of about 2 min prior to injections 2 and 3. Significant sedimentation of 90 μm PS particles could be visually observed within 30 to 60 s as supported by the high sedimentation speed in Table 2.

The modified TOC measurement procedure was then successfully applied to 10, 30, 45, and 90 μm PS particles with recoveries ranging from 79.2 up to 113.6% (Fig. 1a). In this study, the increased variability observed for 30, 45, and 90 μm PS particles was mainly attributed to the fact that these large particles tend to be less homogeneously distributed due to the lower number of particles (Table 2) and despite the mixing step in the syringe prior to injection. As a result, more variation in solid content was to be expected in replicate injections. In particular, in the case of the 30 µm PS particles, the sample was in poor condition with visual signs of particle agglomeration, having been open and used extensively for over a year prior to analysis by the modified TOC method, which likely contributed to higher variability. In comparison, 45 and 90 μm PS particles were freshly purchased and opened prior to analysis and the variability was below 15% (Fig. 1a). Another source of variability in TOC measurements, as discussed by Schmidtmann and Peiffer (2024) [9], may be related to the difficulty of completely burning and oxidizing large PS particles into CO_2_. However, while Schmidtmann and Peiffer (2024) attributed their low recovery for 6 μm PS particles (i.e., 24.5%) using a standard NPOC measurement protocol to incomplete oxidation of the particles to CO_2_, in this study we had no major difficulty in achieving satisfactory recoveries ranging from 80 to 120% for PS particles up to 90 μm using the modified TOC measurement procedure and the high sensitivity catalyst.

The method was not tested with PS particles above 90 µm. However, it is very likely that even the modified TC measurement procedure may fail for larger particles. Indeed, as described in Table 2, in the case of 90 µm PS particles, about 34 particles are injected onto the catalyst with the modified TC procedure. Analyzing larger particles (e.g., 150 or 300 µm PS particles) will require lowering again the number of particles injected to avoid detector saturation down to a point where the number of injected particles will not be statistically sufficient (Table 2) and the modified method may ultimately fail as well. That being said, particles above 90 µm can be observed with the naked eye and can therefore easily be counted and sized by optical microscopy or alternative methods including solid-based TOC.

#### Determination of particle content in other types of polymer-based standard suspensions

Figure 1b shows that consistent recoveries (i.e., 86.2 to 126.2%) and low variability were obtained for the polyethylene (gold-doped PE), polypropylene (gold-doped PP), polyethylene terephthalate (PET), and polyvinyl chloride (gold-doped PVC) particles for which the expected solid contents were measured in-house (Table S4b). It should be noted that the measurement of the expected solid content (i.e., dry weight/wet weight) was hindered by the limited amount of sample available and the low solid content (ca. 0.2% wt from the JRC supplier data corresponding to 100 μg of solid residue per 50 mg of wet sample). However, as mentioned previously, the recovery results were quite consistent and confirmed the low solid content for these PE, PP, PET, and PVC particle suspensions. The high recovery value obtained for PVC (126.2%) was attributed to the possible presence of other sources of carbon in the stock suspensions including bacterial growth. Indeed, these particles were synthesized in June 2020 and stored as aqueous suspension until TOC analysis in 2024.

TOC was measured in the four cellulose samples, after appropriate gravimetric dilution and following dispersion with the probe sonicator (applying 7 kJ in total). The cellulose concentrations obtained from TOC measurements were fully in line with the expected concentrations reported in the CoAs. The recoveries, defined as the ratio between the concentrations obtained from TOC and from CoAs, ranged from 96.3 to 102.4% (Fig. 1b and Table S5).

#### Determination of micro- and nanoplastic content and recovery in environmental water samples

Ultrapure water and environmental surface water samples collected from Lake Maggiore and the Variola River were analyzed by TOC before and after spiking with PS 50 nm, representing the nanoplastic fraction, and PS 1000 nm, representing the lowest size for microplastic fraction (Table 3—Condition 1 vs. 3). Then, the nanoplastic fraction (PS 50 nm) was separated from the microplastic fraction (PS 1000 nm) by means of a filtration step at 0.45 µm and analyzed by TOC in order to evaluate the recovery of the nanoplastic fraction (Table 3—Condition 2 vs. 4). The natural TOC content of the lake water was about four times higher than that of the river water both before (Table 3—Condition 1) and after filtration (Table 3—Condition 2). The difference between the unfiltered and filtered TOC content for the unspiked environmental samples was not statistically significant due to high measurement variability; however, the TOC values are consistent with dissolved organic carbon (Table 3—Condition 2) being lower than total organic carbon (Table 3—Condition 1). PS recovery in spiked environmental surface water samples was evaluated against the results obtained in ultrapure water as a reference after ensuring that the filtration step was efficient in retaining PS 1000 nm (retention rate > 94%) and rejecting PS 50 nm (rejection rate > 99%). As shown in Table 3, the PS recoveries for the spiked lake water were very high (≥ 95%) both before and after removal of the microplastic fraction. PS recovery was also high (≥ 90%) for the spiked river water before filtration but decreased to 70% after removal of the microplastic fraction. This lower recovery observed for the nanoplastic fraction in the river water could be an indication that some PS 50 nm particles interacted with or due to other components present in the river water (e.g., via hetero-aggregation) and were not able to pass freely through the filtration membrane. Indeed, organic species (e.g., humic acids, fulvic acids) can contribute to the stabilization of nanoplastics, while divalent cations (e.g., Mg^2+^, Ca^2+^) and metal oxides (e.g., iron oxides) can increase (hetero-)aggregation [22–24]. In the present case, compared to the lake water, the river water contained less organic carbon (TOC = 0.17 vs. 0.75 mg L^−1^) but more inorganic carbon (TIC = 14.6 vs. 10.3 mg L^−1^) and overall more divalent cations, especially Mg^2+^ (Table 3 and Table S2). The different water compositions (organic and inorganic species) may affect the (hetero-)aggregation phenomena of nanoplastics differently. It should be noted that while this study focused on commercially available PS particles, real-world environmentally aged and weathered micro- and nanoplastics (MNPs) may add another level of complexity as they may present significantly different behaviors than pristine particles. Changes in physicochemical properties such as increased adsorption or decreased hydrophobicity can be expected but remain difficult to predict [25]. Nevertheless, TOC remains a valid tool even for real-world MNPs tracking and quantification during sample preparation.

**Table 3:** TOC measurements in triplicate (average ± standard deviation) and polystyrene particle recoveries before and after filtration at 0.45 µm in ultrapure water and environmental water samples spiked with PS 1000 nm and PS 50 nm

		TOC^a^ (mg L^−1^) [PS recovery] (%)		
	Conditions (*n* = 3)	UPW (reference^c^)	Lake Maggiore	Variola River
Unspiked samples	**(1) Unfiltered**	0.028 ± 0.003	0.872 ± 0.098	0.202 ± 0.139
	**(2) Filtered 0.45 µm**	0.094 ± 0.002	0.750 ± 0.070	0.167 ± 0.046
Spiked^b^ samples	**(3) Unfiltered**	1.712^c^ ± 0.014 [100 ± 2]	1.637 ± 0.131 [96 ± 8]	1.538 ± 0.325 [90 ± 19]
	**(4) Filtered 0.45 µm**	0.830^c^ ± 0.004 [100 ± 1]	0.788 ± 0.223 [95 ± 27]	0.577 ± 0.265 [70 ± 32]

^a^For conditions (3) and (4), native TOC contribution of unspiked sample (unfiltered or filtered) was subtracted, respectively

^b^Unfiltered water spiked with 1 mg L^−1^ PS 1000 nm and 1 mg L^−1^ PS 50 nm (theoretically equivalent to (1 + 1) × 92.26%C = 1.8452 mg L^−1^ organic carbon addition)

^c^Reference TOC values for PS recoveries calculations in conditions (3) and (4), respectively

### Assessment of micro- and nanoplastic fractionation efficiency following centrifugal fractionation

**Fig. 2:**
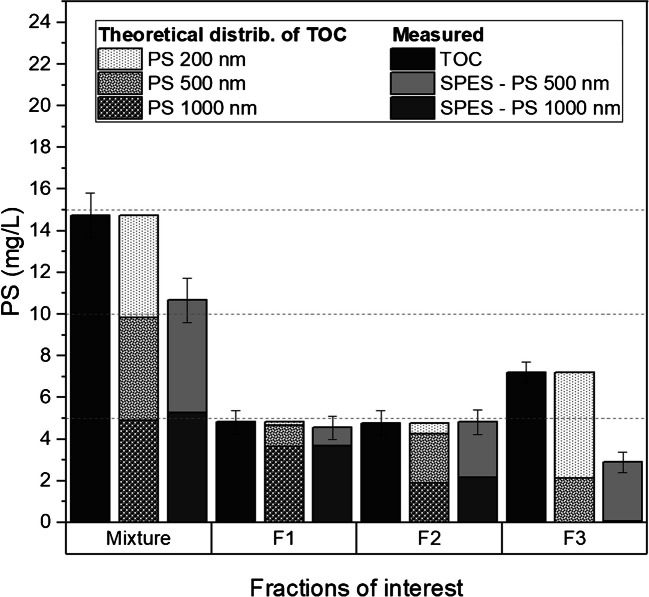
Comparison of the mass concentration obtained by TOC and SPES as well as theoretical distribution of the particles in each fraction according to Stokes’ law and the actual g-force gradient experienced by the samples. Horizontal lines at 5, 10, and 15 mg L^−1^ added as visual guides

TOC and SPES were employed to monitor and quantify the recovery of nano- and microplastics during centrifugal separation using an equimassic mixture of three commercially available PS beads. Figure 2 presents a comparison of the mass-based concentrations obtained via both techniques as well as the theoretical distribution of the particles in each fraction according to Stokes’ law and the actual g-force gradient experienced by the samples. The recovery obtained by TOC for the starting mixture (i.e., before centrifugation) was 98.3 ± 7.1%, showing good agreement with the solid contents reported on the CoAs. The SPES analysis of the same starting mixture showed recoveries of 108.3 ± 10.2% for PS 500 nm and 105.0 ± 8.2% for PS 1000 nm. SPES is not able to detect PS 200 nm, but the good agreement between theoretical and measured values of TOC, SPES 500 nm and SPES 1000 nm resulted in a recovery of 81.5 ± 42.6% for PS 200 nm by subtraction (as PS 200 nm = TOC − SPES 500 nm − SPES 1000 nm). The addition of uncertainties from TOC and SPES values resulted in a high uncertainty budget for the PS 200 nm recovery.

It is important to note that the centrifugation times to obtain a cutoff of 1000 nm for C1 and 500 nm for C2 were derived from Stokes’ law by taking into account the RCF values (2500 RCF for C1 or 4000 RCF for C2) and the temperature (20 °C) set on the centrifuge screen, the height of the liquid suspension in the sample tube (60 mm), the densities of PS particles (1.05 g cm^−3^) and water (1 g cm^−3^), and the viscosity of water at 20 °C (1.0016 mPa s). However, the RCF values set on the centrifuge screen (2500 RCF or 4000 RCF) are only valid at the outer diameter of the rotor (Fig. S2S2). In fact, a g-force gradient is expected from the center of the rotor (virtually 0 RCF) to the outer limit of the rotor (2500 RCF or 4000 RCF). This in turn means that the sample tube which is located somewhere in between the center and the outer limit of the rotor, will also experience a g-force gradient. After measuring several dimensions of the rotor, sample holder, and sample tube, it was possible to calculate the actual g-force gradient in the fluid column. For C1, the g-force gradient ranged from 1042 RCF at the top of the liquid (i.e., closer to the rotor center) to 1976 RCF at the bottom of the sample tube. Taking into account the centrifugation time used (14 min), the effective cutoff was calculated to be 1341 nm at 60 mm. For C2, the g-force gradient ranged from 1668 RCF at the top of the liquid to 3161 RCF at the bottom of the liquid. Considering the centrifugation time used (30 min), the effective cutoff was calculated to be 724 nm at 60 mm.

Following the first centrifugation (C1: 2500 RCF, 14 min, 1000 nm theoretical cutoff and 1341 nm effective cutoff), TOC analysis of pellet F1 (Fig. 2—F1), which was expected to contain primarily microparticles, showed a total PS concentration of 4.8 ± 0.6 mg L^−1^. SPES analysis of pellet F1 confirmed that 76.0% of this concentration was due to PS 1000 nm particles and 18.3% to PS 500 nm particles in line with the theoretical values derived from Stokes’ law of 75.1% and 21.3%, respectively.

Subsequently, TOC analysis of pellet F2 (Fig. 2—F2) and supernatant F3 (Fig. 2—F3) after the second centrifugation (C2: 4000 RCF, 30 min, 500 nm theoretical cutoff and 724 nm effective cutoff) revealed total PS concentrations of 4.8 ± 0.6 mg L^−1^ and 7.2 ± 0.5 mg L^−1^, respectively. SPES analysis of pellet F2 indicated that 55.7% of the concentration in pellet F2 was attributed to PS 500 nm particles, while PS 1000 nm accounted for 45.2% of the concentration. These values are in line with the theoretical values of 49.5% for PS 500 nm and 39.9% for PS 1000 nm according to Stokes’ law. The presence of PS 1000 nm in the pellet F2 was expected since the sedimentation of these particles was not quantitative during the first centrifugation step (C1). However, SPES analysis demonstrated that PS 1000 nm particles were practically absent from supernatant F3 with a concentration < 0.1 mg L^−1^ in line with the effective cutoff for C2 (i.e., 724 nm). According to SPES, PS 500 nm represented a concentration of 2.8 ± 0.5 mg L^−1^ in supernatant F3, in line with a theoretical value of 2.12 mg L^−1^. The remaining 4.3 mg L^−1^ detected in supernatant F3 accounted for 87.4% of the PS 200 nm initially introduced into the mixture, in line with the theoretical value of 84.8%. Overall, the sum of F1 + F2 + F3 yielded recoveries of 113.8 ± 13.9% for TOC, 112.5 ± 15.5% for SPES 1000 nm, and 117.3 ± 15.5% for SPES 500 nm. By subtraction, these results would leave a theoretical overall recovery of 90.5 ± 66.3% for PS 200 nm. Once again, the addition of uncertainties from TOC and SPES values resulted in a high uncertainty budget for the PS 200 nm recovery. However, this overall recovery is consistent with the one calculated by subtraction from the initial mixture (i.e., 81.5 ± 42.6%) for PS 200 nm.

The combination of TOC and SPES measurements allowed the tracking and quantification of three distinct populations of PS particles during successive centrifugation steps of the initial mixture. The results demonstrated the complementarity of these techniques when dealing with polydisperse mixtures. In fact, in the present case, SPES was not able to detect PS 200 nm, whereas TOC allowed the estimation of this fraction by subtraction before and after two consecutive centrifugation steps. In addition, this combination of analyses allows fine-tuning of centrifugation conditions (speed/time) according to requirements. For example, for the first centrifugation (C1), a theoretical cutoff of 1000 nm was used, resulting in a small fraction of PS 1000 nm population remaining in the supernatant (ca. 20%) and a small fraction of PS 500 nm population present in the pellet F1 (ca. 20%). Another theoretical cutoff can be used such as 900 nm, which will increase the recovery of PS 1000 nm particles in the pellet (closer to 100%), but at the same time, as the suspension is homogeneous in the vial height when the centrifugation starts (vs. line-start centrifugation), it will increase the amount of PS 500 nm particles in the pellet. In conclusion, this method is useful for finding a balance in the centrifugal conditions when the intention is to separate nanoparticles from microparticles.

### TOC as a tool for assessing filtration and AF4 recoveries of nanocellulose suspensions

#### Filtration and AF4 fractionation recoveries

**Fig. 3:**
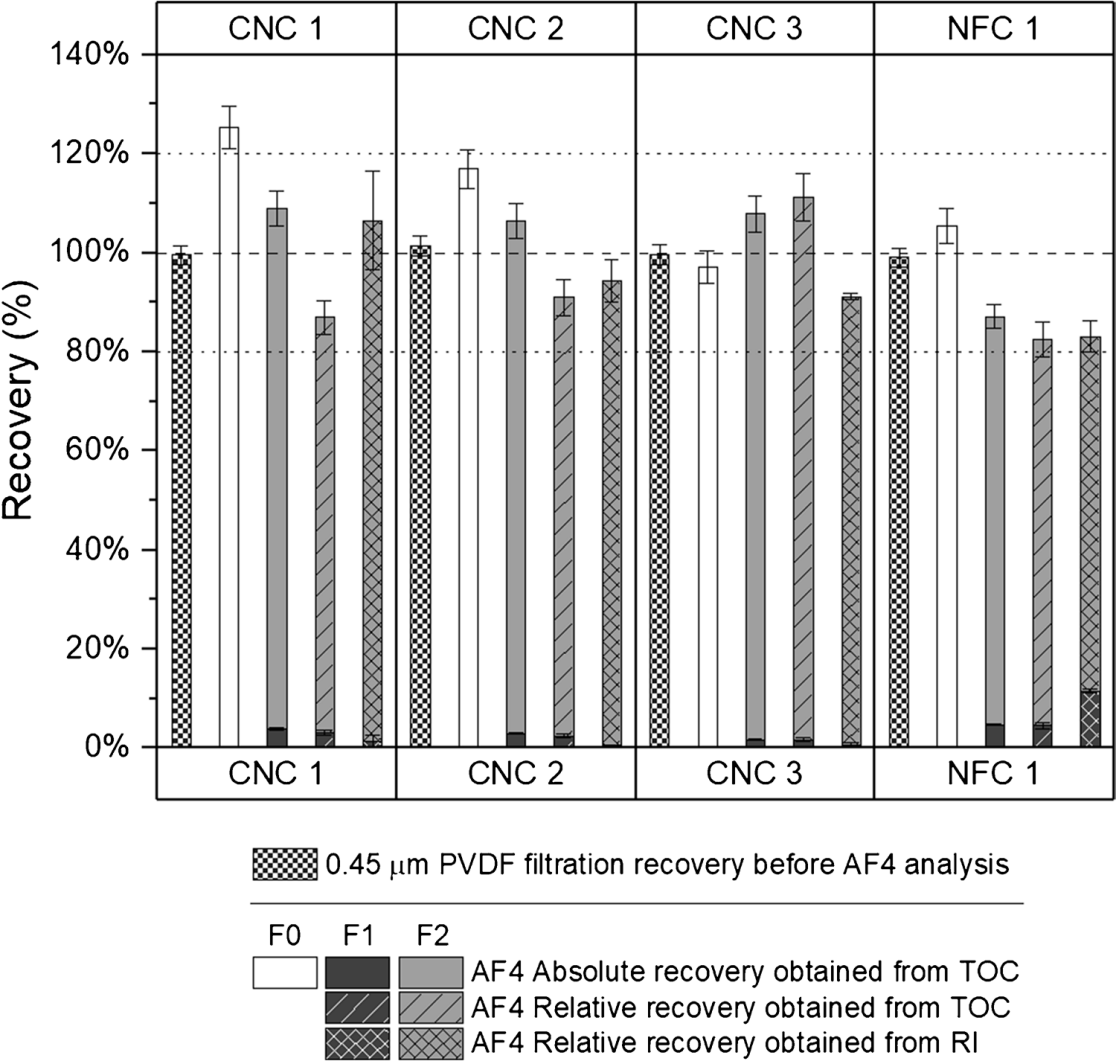
Filtration (0.45 μm PVDF), absolute and relative AF4 recoveries measured by TOC and/or RI for different types of nanocellulose and cellulose nanofiber materials in different AF4 fractions. Horizontal lines at 80%, 100%, and 120% added as visual guides

The four diluted and well-dispersed cellulose suspensions were filtered through a 0.45-μm PVDF syringe filter and the filtrate was analyzed by TOC after appropriate gravimetric dilution. Filtration recoveries ranged from 99.0 to 101.4% (Fig. 3 and Table S5). These very good recoveries indicated the absence of sample loss during filtration (i.e., recovery significantly < 100%). Possible carbon contamination from the filtration step itself was ruled out by analysis of filtration blanks where no carbon was detected.

#### AF4 fractions (fractionation recovery)

AF4 fractionation recovery is defined by ISO as “the ratio of the mass eluted during fractionation to the mass initially injected, expressed as a percentage” [26]. It can be routinely assessed by the ratio of the UV or RI peak area produced by a sample with crossflow (i.e., actual conditions for particle separation into different size fractions) and without crossflow (and without focus flow during the focusing step). In fact, in absence of a separating force field, recovery is assumed to be 100% and the peak area can be used as “reference” area value. In this regard, once filtered and further diluted to 0.3% wt, the four cellulose samples were injected into the AF4-MALS-RI system with and without crossflow. The fractograms show one main population F2 (one broad peak) and a small population F1 (in terms of size and concentration) at the void time (1 min). The particles are well fractionated with size (radius of gyration) varying from about 20 to 60 nm (Fig. S1).

TOC was measured, after appropriate gravimetric dilution, in two main fractions (i.e., F1 and F2) collected with crossflow and in the single fraction (i.e., F0) collected without crossflow. The recovery of the AF4 fractionation was then calculated in two different ways:

(i) “Relative” recovery was the ratio of TOC measured in F1 or F2 and TOC measured in F0 while;
(ii) “Absolute” recovery was the ratio of TOC measured in F1 or F2 and the TOC measured in the injected sample (i.e., TOC measured in 50 μL of 0.3% cellulose suspension).

The measurement of the relative recovery with TOC is similar in its principle to the recovery assessed by the RI signal in that it requires the comparison of two injections of the sample, one with and one without application of the separation force field. On the other hand, if the mass of the injected analytes is known, then only one injection of the sample with the force field is required to measure the absolute recovery as “the ratio of the mass eluted during fractionation to the mass initially injected” in line with the primary ISO definition of AF4 recovery. Figure 3 presents the recoveries obtained by TOC (absolute and relative) and RI for fractions F1 and F2. The total (i.e., F1 + F2) absolute recoveries obtained from TOC were in the range of 87 to 109% while total relative recoveries were in the range of 83 to 111%. Both relative and absolute recoveries were therefore in the range considered acceptable in ISO technical specification for AF4 (i.e., ≥ 70%) [26]. In comparison, RI recoveries were in the range of 83 to 106% and therefore in line with both absolute and relative recoveries obtained by TOC.

It should be noted that the relative TOC recoveries were significantly lower than the absolute TOC recoveries for CNC 1 and 2. This was attributed to the unexpectedly high absolute TOC recoveries measured for F0 (i.e., 125% for CNC 1 and 117% for CNC 2), which in turn influenced the calculation of the relative TOC recoveries for CNC 1 and 2, as F0 served as a “reference” value.

The relative uncertainties of the absolute TOC recoveries were estimated to be ≤ 5.0% for F1, and ≤ 3.4% for F2 and F0, while for the relative TOC recoveries they were estimated to be ≤ 23.3% for F1 and ≤ 4.5% for F2. This is mainly due to a higher uncertainty budget in the case of relative recovery. Indeed, the uncertainty on F0 was taken into consideration.

These results demonstrate that TOC can provide a universal (i.e., non-specific) measure of the absolute recovery of a carbon-based particulate material after AF4 fractionation. This could easily be transferred to other types of field flow fractionation techniques (e.g., centrifugal, electrical, thermal). However, a major limitation of this strategy could be the use of an organic compound (e.g., surfactant, organic buffer) in the mobile phase at a concentration that cannot be easily and confidently subtracted from the analyte signal. Another limitation, depending on the AF4 method (e.g., selectivity, membrane cutoff), would be the presence of significant amounts of small organic molecules or macromolecules in the initial sample that could contribute to the TOC measurements in the AF4 fractions or in the initial sample, but may also give a signal on the RI detector.

## Conclusion

TOC is not a chemically specific detector, which means that it will detect any source of carbon, including micro- and nano-sized particulate organic matter. Thus, when focusing on the analysis of synthetic and natural polymers, limitations arise mainly from interfering carbon sources such as organic molecules (e.g., humic acids, proteins, surfactants, solvents) and organisms (e.g., bacteria, algae). These limitations can be overcome by developing appropriate sample preparation procedures and analytical steps enabling a clearer understanding of what we are measuring. In this regard, TOC can be a powerful tool for quickly evaluating the efficiency of sample preparation methods.

Liquid-based TOC measurements have proven effective for determining the stock concentration of various polymer-based particle suspensions in ultrapure water samples. Good agreement was found between the reported solid content and standard TOC measurements across various polymer types and sizes. However, for larger particles (> 10 μm), recovery decreased significantly, revealing size-related limitations attributed to faster particle sedimentation and necessitating modifications and optimization of the standard TOC procedure to overcome this limitation. These adjustments included increasing the injection volume, calibration range, and addressing sedimentation issues, ultimately resulting in improved recoveries for particles up to 90 μm. The modified TOC procedure was not tested with PS particles larger than 90 µm, but a theoretical approach suggests that even the modified TC measurement procedure is likely to fail for particles larger than 150 µm, for which solid-based TOC may be more appropriate.

Liquid-based TOC measurements also proved useful in evaluating the efficiency of particle separation methods such as filtration, centrifugation, and AF4. The efficiency of centrifugation and AF4 fractionation for size separation was confirmed with TOC providing a quick estimate of recovery efficiency. Especially for the absolute recovery of AF4 fractionation, taking into account all potential sources of loss in the system (inside and outside the separation channel), the results are in line with the ISO technical specification. Additionally, the combination of liquid-based TOC with other preparative and analytical techniques, such as SPES, might improve the characterization and quantification of micro- and nanoplastics in more complex matrices.

In the environmental field, measurements of TOC and TIC provide important information on the composition of the water sample. This information leads to the application of appropriate analytical methods to identify and quantify target particles. In fact, the application of TOC to environmental samples allowed to quickly suspect possible interactions, such as hetero-aggregation, between the nanoplastic fraction and the natural constituents present in the freshwater. In the same way, TOC can be used to investigate the effects of sample treatment (e.g., sample pre-concentration methods, UV and H_2_O_2_ oxidation-digestion to remove or reduce organic carbon content, sample pre-acidification to remove carbonates) on environmental water samples of different origin and with different organic content. In this context, future research is needed to further compare the two TOC quantification methods (NPOC vs. TC-TIC) and to assess their applicability and limitations to different types of weathered microplastic samples.

## Supplementary Information

Below is the link to the electronic supplementary material.

Supplementary file1 (PDF 733 KB)

Supplementary file2 (XLSX 13 KB)

## References

[1] Enders K, Käppler A, Biniasch O, Feldens P, Stollberg N, Lange X, Fischer D, Eichhorn K-J, Pollehne F, Oberbeckmann S, Labrenz M. Tracing microplastics in aquatic environments based on sediment analogies. Sci Rep. 2019;9(1):15207. 10.1038/s41598-019-50508-2. 31645581 10.1038/s41598-019-50508-2 PMC6811616

[2] Ling SD, Sinclair M, Levi CJ, Reeves SE, Edgar GJ. Ubiquity of microplastics in coastal seafloor sediments. Mar Pollut Bull. 2017;121(1):104–10. 10.1016/j.marpolbul.2017.05.038. 28571629 10.1016/j.marpolbul.2017.05.038

[3] Li P, Lai Y, Li Q, Dong L, Tan Z, Yu S, Chen Y, Sharma VK, Liu J, Jiang G. Total organic carbon as a quantitative index of micro- and nano-plastic pollution. Anal Chem. 2022;94(2):740–7. 10.1021/acs.analchem.1c03114. 34974702 10.1021/acs.analchem.1c03114

[4] Zheng R, Li Q, Li P, Li L, Liu J. Total organic carbon content as an index to estimate the sorption capacity of micro- and nano-plastics for hydrophobic organic contaminants. Chemosphere. 2023;313: 137374. 10.1016/j.chemosphere.2022.137374. 36435320 10.1016/j.chemosphere.2022.137374

[5] Hong Y, Oh J, Lee I, Fan C, Pan S-Y, Jang M, Park Y-K, Kim H. Total-organic-carbon-based quantitative estimation of microplastics in sewage. Chem Eng J. 2021;423: 130182. 10.1016/j.cej.2021.130182.

[6] Gonzales de Vega R, Goyen S, Lockwood TE, Doble PA, Camp EF, Clases D. Characterisation of microplastics and unicellular algae in seawater by targeting carbon via single particle and single cell ICP-MS. Anal Chim Acta. 2021;1174: 338737. 34247735 10.1016/j.aca.2021.338737

[7] Kim J-W, Lim H-B, Jang J-Y, Shin H-S. Sludge-based candidate reference materials for enhanced quality control of particulate processes in total organic carbon analysis for wastewater1. Chemosphere. 2024;352: 141458. 10.1016/j.chemosphere.2024.141458. 38364920 10.1016/j.chemosphere.2024.141458

[8] Lee H-S, Hur J, Hwang Y-H, Shin H-S. A novel procedure of total organic carbon analysis for water samples containing suspended solids with alkaline extraction and homogeneity evaluation by turbidity. Int J Environ Res Public Health. 2020;17(11):3901. 32486395 10.3390/ijerph17113901 PMC7311996

[9] Schmidtmann J, Peiffer S. A rapid method to quantify sub-micrometer polystyrene particles in aqueous model systems by TOC analysis. Microplast Nanoplast. 2024;4(1):3. 10.1186/s43591-024-00080-y.

[10] Water analysis. Guidelines for the determination of total organic carbon (TOC) and dissolved organic carbon (DOC), EN 1484:1997.

[11] Directive (EU) 2020/2184 of the European Parliament and of the Council of 16 December 2020 on the quality of water intended for human consumption (recast), Directive (EU) 2020/2184, European Parliament, 2020. http://data.europa.eu/eli/dir/2020/2184/oj. Accessed 20 Jan 2025.

[12] Proposal for a directive of the European parliament and of the council concerning urban wastewater treatment (recast), COM/2022/541, European Commission, 2022. https://eur-lex.europa.eu/legal-content/EN/TXT/?uri=celex:52022PC0541. Accessed 20 Jan 2025.

[13] Lee H-S, Hur J, Lee M-H, Brogi SR, Kim T-W, Shin H-S. Photochemical release of dissolved organic matter from particulate organic matter: Spectroscopic characteristics and disinfection by-product formation potential. Chemosphere. 2019;235:586–95. 10.1016/j.chemosphere.2019.06.127. 31276871 10.1016/j.chemosphere.2019.06.127

[14] Water quality — Vocabulary, ISO 6107:2021.

[15] Cassano D, La Spina R, Ponti J, Bianchi I, Gilliland D. Inorganic species-doped polypropylene nanoparticles for multifunctional detection. ACS Appl Nano Mater. 2021;4(2):1551–7.

[16] Cassano D, Bogni A, La Spina R, Gilliland D, Ponti J. Investigating the cellular uptake of model nanoplastics by single-cell ICP-MS. Nanomaterials. 2023;13(3):594. 36770555 10.3390/nano13030594 PMC9920308

[17] Guo L, Santschi PH. Ultrafiltration and its applications to sampling and characterisation of aquatic colloids. In: Environmental colloids and particles. 2006. pp 159–221. 10.1002/9780470024539.ch4.

[18] Schuck P. Sedimentation coefficient distributions of large particles. Analyst. 2016;141(14):4400–9. 10.1039/C6AN00534A. 27196374 10.1039/c6an00534a PMC4931980

[19] Potenza M, Villa S, Sanvito T, Albani S, Delmonte B, Maggi V. Dust optical properties in antarctic ice cores: application of the Single Particle Extinction and Scattering (SPES) method. In: EGU General Assembly Conference Abstracts. 2015;10937. https://meetingorganizer.copernicus.org/EGU2015/EGU2015-10937.pdf. Accessed 20 Jan 2025.

[20] Villa S, Sanvito T, Paroli B, Pullia A, Delmonte B, Potenza M. Measuring shape and size of micrometric particles from the analysis of the forward scattered field. J Appl Phys. 2016;119. 10.1063/1.4953332.

[21] Mukherjee A, Hackley VA. Separation and characterization of cellulose nanocrystals by multi-detector asymmetrical-flow field-flow fractionation. Analyst. 2018;143(3):731–40. 10.1039/C7AN01739A. 29322138 10.1039/c7an01739a PMC6057617

[22] Reynaud S, Aynard A, Grassl B, Gigault J. Nanoplastics: from model materials to colloidal fate. Curr Opin Colloid Interface Sci. 2022;57: 101528. 10.1016/j.cocis.2021.101528.

[23] Pradel A, Catrouillet C, Gigault J. The environmental fate of nanoplastics: what we know and what we need to know about aggregation. NanoImpact. 2023;29: 100453. 10.1016/j.impact.2023.100453. 36708989 10.1016/j.impact.2023.100453

[24] Schmidtmann J, Elagami H, Gilfedder BS, Fleckenstein JH, Papastavrou G, Mansfeld U, Peiffer S. Heteroaggregation of PS microplastic with ferrihydrite leads to rapid removal of microplastic particles from the water column. Environ Sci Process Impacts. 2022;24(10):1782–9. 10.1039/D2EM00207H. 36001017 10.1039/d2em00207h

[25] Gigault J, El Hadri H, Nguyen B, Grassl B, Rowenczyk L, Tufenkji N, Feng S, Wiesner M. Nanoplastics are neither microplastics nor engineered nanoparticles. Nat Nanotechnol. 2021;16(5):501–7. 10.1038/s41565-021-00886-4. 33927364 10.1038/s41565-021-00886-4

[26] Nanotechnologies - Analysis of nano-objects using asymmetrical-flow and centrifugal field-flow fractionation, ISO/TS 21362:2018.

