# Supplementary material for: Total organic carbon (TOC): a simple tool for assessing micro(nano)plastics and nanocellulose recovery during size-based fractionation
Source: Anal Bioanal Chem. 2025 May 19;417(14):2983–96. doi: 10.1007/s00216-025-05812-4 (PMC12103321; doi:10.1007/s00216-025-05812-4)
Supplement: Supplementary file 1 — Supplementary file1 (PDF 733 KB) [file 216_2025_5812_MOESM1_ESM.pdf]

Total Organic Carbon (TOC): a simple tool for assessing micro(nano)plastics and nanocellulose recovery during size-based fractionation

Guillaume Bucher[1], Gabriella F. Schirinzi[1], Chiara Verra[1], Hind El Hadri[1], Otmar Geiss[1], Douglas Gilliland*[1]

[1]European Commission, Joint Research Centre (JRC), Ispra, Italy

*Corresponding Author

Supplementary Information

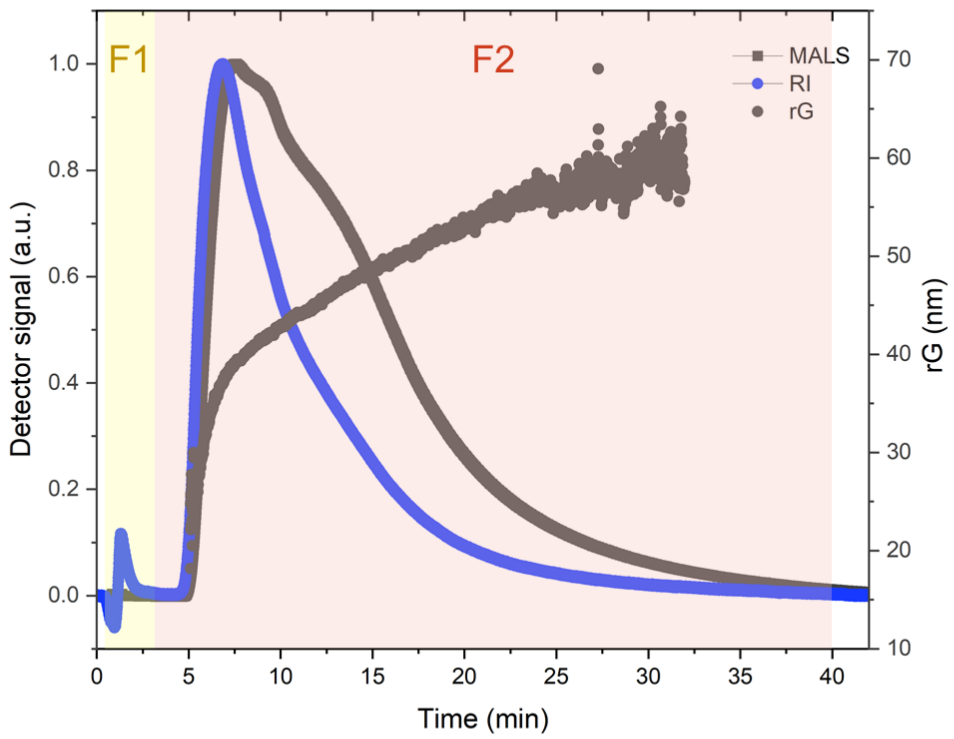

Fig. S1 Typical fractogram of a crystalline nanocellulose sample during AF4 fractionation with MALS, rG and RI signals. The collection times of fractions F1 and F2 are shown in the background in yellow and orange, respectively.

Table S2. pH, total inorganic carbon (TIC) and concentrations of 39 elements in environmental surface water samples collected from Lake Maggiore (Ispra, Italy, Coordinates 45°48'23.8"N - 8°36'23.7"E) and the Variola River (Monte Ossolano, Italy, Coordinates 46°07’31.2”N - 8°13’29.3”E).

**Element Concentrations ($\mu g\ L^{-1}$)**	**Lake Maggiore**	**Variola River**
	**pH 7.84**	**pH 8.03**
	**TIC 10.3 mg L^{-1}**	**TIC 14.6 mg L^{-1}**
**Li**	0.909	<LOD (0.6)
**Be**	<LOD (0.4)	<LOD (0.4)
**B**	2.8	<LOD (0.7)
**Na**	2735	798
**Mg**	3373	6576
**Al**	5.93	4.22
**Si**	3718	6861
**Cl**	3542	896
**K**	1445	409
**Ca**	13721	12984
**Sc**	0.010	<LOD (0.007)
**Ti**	0.125	<LOD (0.1)
**V**	0.150	0.184
**Cr**	0.079	1.691
**Mn**	0.876	0.099
**Fe**	5.065	0.590
**Co**	0.016	0.012
**Ni**	0.739	3.396
**Cu**	0.766	0.574
**Zn**	<LOD (0.014)	0.103
**As**	1.283	0.222
**Se**	0.206	0.247
**Rb**	2.057	0.562
**Sr**	200	181
**Y**	0.017	0.006
**Zr**	0.006	0.005
**Mo**	1.082	2.467
**Ru**	<LOD (0.004)	<LOD (0.004)
**Rh**	<LOD (0.001)	<LOD (0.001)
**Pd**	0.017	0.020
**Ag**	0.002	<LOD (0.001)
**Cd**	<LOD (0.011)	0.016
**In**	<LOD (0.002)	<LOD (0.002)
**Sn**	0.015	0.040
**Ba**	12.4	3.9
**Eu**	<LOD (0.001)	<LOD (0.001)
**Au**	0.015	0.015
**Pb**	0.018	0.089
**Bi**	<LOD (0.001)	<LOD (0.001)

Table S3. AF4 parameters for nanocellulose fractionation

AF4 parameters	
**Membrane**	Regenerated cellulose 10 kDa
**Channel**	Short channel with 350 µm spacer
**Temperature**	25.0 ± 0.1 °C (channel and detectors)
**Eluent**	1 mM NaCl
**Detector flow**	0.5 mL min^{-1}
**Cross flow**	0.8 mL min^{-1}
**Focus flow**	2 mL min^{-1}
**Elution method**	2 min elution + cross flow<br>2 min focus<br>3 min focus + injection<br>3 min focus<br>60 min elution + cross flow<br>5 min elution (no cross flow)

Table S4a. Expected and TOC-derived solid content for different PS micro- and nano-particle standards

	Particle characteristics		TOC measurement procedure	Solid Content (%wt)			
Sample Name	Nominal Size (nm)	Polymer Type		Expected	Source	Measured by TOC	Recovery (%) as Measured/Expected
PS 50 nm	50	PS	Standard	2.6%	CoA	2.4%	92.6% ± 0.7%
PS 100 nm	100	PS	Standard	2.6%	CoA	2.7%	104.8% ± 0.3%
PS 200 nm SA	200	PS	Standard	2.2%	Measured[a]	2.6%	118.0% ± 1.1%
PS 200 nm Poly	200	PS	Standard	2.6%	CoA	2.7%	104.9% ± 0.2%
PS 200 nm Fluo	200	PS	Standard	2.6%	CoA	2.6%	101.2% ± 0.3%
PS 500 nm	500	PS	Standard	2.7%	CoA	2.6%	98.1% ± 0.3%
PS 750 nm	750	PS	Standard	2.7%	CoA	2.7%	100.0% ± 0.7%
PS 1 µm	1000 (1 µm)	PS	Standard	2.6%	CoA	2.6%	100.7% ± 1.5%
PS 8 µm	8000 (8 µm)	PS	Standard	15.8%	Measured[a]	13.9%	87.9% ± 0.3%
PS 10 µm	10000 (10 µm)	PS	Standard	2.3%	Measured[a]	2.0%	85.4% ± 0.8%
			Modified			1.8%	79.2% ± 1.4%
PS 30 µm	30000 (30 µm)	PS	Standard	10.0%	Measured[a]	7.4%	73.4% ± 3.7%
			Modified			11.4%	113.6% ± 27.7%
PS 45 µm	45000 (45 µm)	PS	Standard	2.7%	CoA	1.6%	60.4% ± 10.4%
			Modified			2.9%	107.2% ± 5.3%
PS 90 µm	90000 (90 µm)	PS	Standard	2.5%	CoA	0.14%	5.6% ± 0.03%
			Modified			2.8%	111.6% ± 15.9%

[a]solid content measured in-house as = dry weight of particle residue (drying overnight at 40°C) / wet weight of particle suspension (50 µL of suspension)

Table S4b. Expected and TOC-derived solid content for different types of micro- and nano-particles

	Particle characteristics		%Carbon in the polymer	TOC measurement procedure	Solid Content (%wt)			
Sample Name	Size (nm)	Polymer Type			Expected	Source	Measured by TOC	Recovery (%) as Measured/Expected
Au-PE	≈ 120<br>(ρ = 0.967 g cm^{-3})	PE	85.63%	Standard	0.19%	Measured[a]	0.18%	93.8% ± 1.0%
Au-PP	≈ 120<br>(ρ = 0.962 g cm^{-3})	PP	85.63%	Standard	0.24%	Measured[a]	0.22%	89.7% ± 0.8%
Au-PVC	≈ 120<br>(ρ = 1.435 g cm^{-3})	PVC	38.44%	Standard	0.22%	Measured[a]	0.28%	126.2% ± 0.6%
CNC 1	≈ 7 (diameter)<br>≈ 208 (length)	crystalline nanocellulose	44.45%	Standard	10.6%	CoA	10.21%	96.3% ± 0.9%
CNC 2	≈ 6 (diameter)<br>≈ 169 (length)	crystalline nanocellulose	44.45%	Standard	6.0%	CoA	6.07%	101.2% ± 1.0%
CNC 3	≈ 5 (diameter)<br>≈ 220 (length)	crystalline nanocellulose	44.45%	Standard	6.0%	CoA	5.91%	98.5% ± 1.0%
NFC 1	≈ 7 (diameter)<br>≈ 188 (length)	TEMPO-oxidized cellulose nanofibers	40.92%	Standard	1.0%	CoA	1.024%	102.4% ± 1.0%
PET	< 5000<br>(ρ = 1.4 g cm^{-3})	PET	62.50%	Standard	0.0025%	Measured[a]	0.0021%	86.2% ± 2.9%

[a]solid content measured in-house as = (dry weight of particle residue (drying overnight at 40°C) / wet weight of particle suspension (50 µL of suspension)) – contribution from Au to the mass of particles

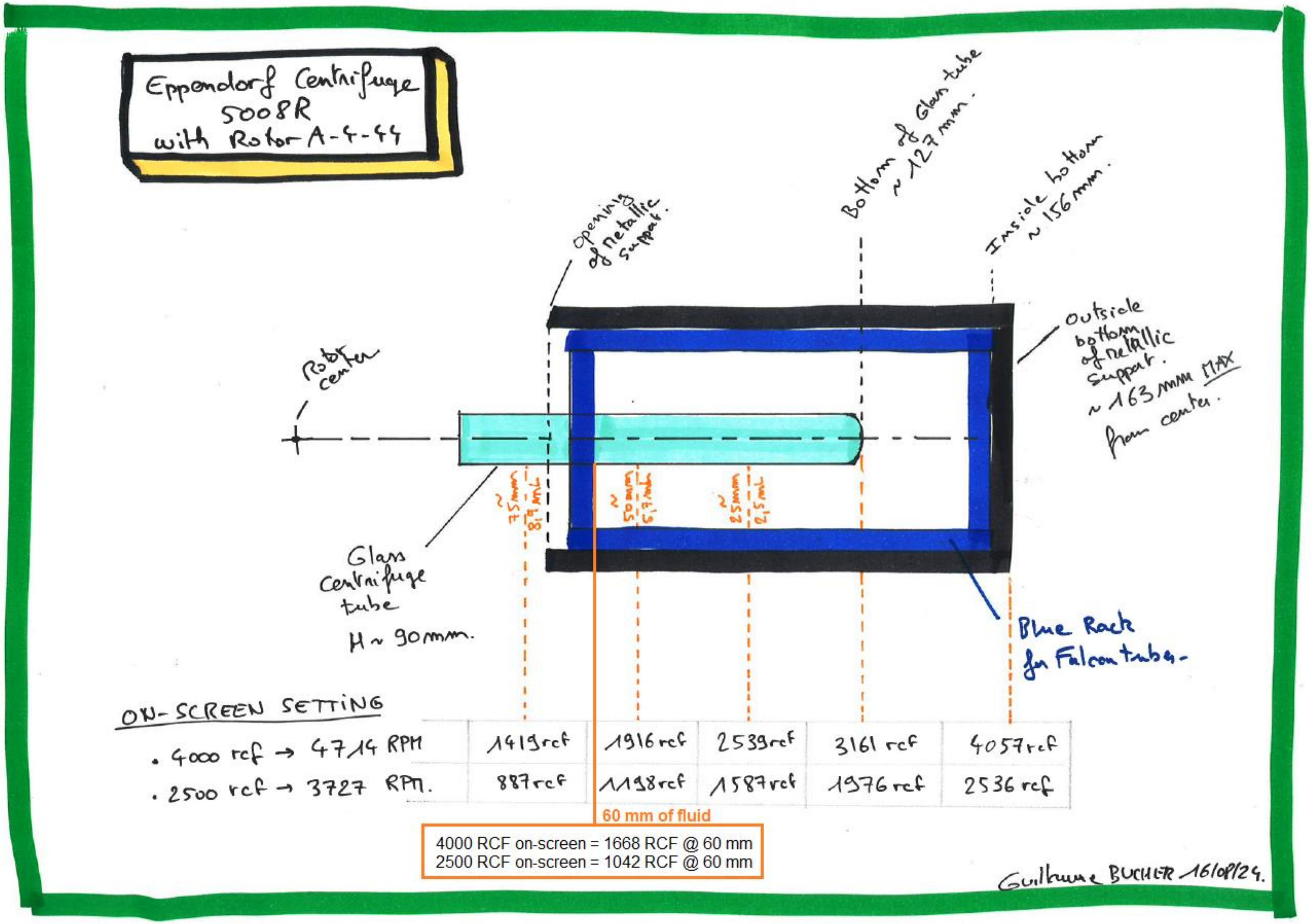

Fig. S2 Diagram (top view) of the A-4-44 rotor to scale, showing the position of the tube containing the sample. The table at the bottom indicates the actual g-force (expressed in RCF) experienced by the sample at various position with a focus at 60 mm when setting 2500 RCF or 4000 RCF on the centrifuge screen.

Table S5. Expected and measured cellulose concentrations in stock suspensions before and after filtration with 0.45 µm PVDF syringe filters.

Material	CNC 1	CNC 2	CNC 3	NFC 1
Cellulose concentration from CoA (% wt)	10.60	6.00	6.00	1.00
Cellulose concentration from TOC before filtration (% wt)	10.21 ± 0.10	6.07 ± 0.06	5.91 ± 0.06	1.024 ± 0.01
Recovery as ratio of TOC vs. CoA (%)	96.3 ± 0.9	101.2 ± 1.0	98.5 ± 1.0	102.4 ± 1.0
Cellulose concentration from TOC after filtration (% wt)	10.16 ± 0.10	6.15 ± 0.06	5.90 ± 0.06	1.015 ± 0.01
Recovery after filtration 0.45 µm PVDF (%)	99.5 ± 2.0	101.4 ± 2.0	99.7 ± 2.0	99.0 ± 2.0
